# Evaluation the frequency of factor V Leiden mutation in pregnant women with preeclampsia syndrome in an Iranian population

**Published:** 2012-01

**Authors:** Samieh Karimi, Majid Yavarian, Azadeh Azinfar, Minoo Rajaei, Maryam Azizi Kootenaee

**Affiliations:** 1 Department of Obstetrics and Gynecology, Hormozgan Fertility and Infertility Research Center, Shariati Hospital, Hormozgan University of Medical Sciences, Bandar Abbas, Iran.; 2 Department of Human Molecular and Clinical Genetics, Shiraz University of Medical Sciences, Shiraz, Iran.

**Keywords:** *Preeclampsia*, *Factor V Leiden mutation*, *Obstetric complication*, *Iran*

## Abstract

**Background: **Role of genetic factors in etiology of preeclampsia is not confirmed yet.

**Objective**: Gene defect frequency varies in different geographic areas as well as ethnic groups. In this study, the role of factor V Leiden mutation in the pathogenesis of preeclampsia syndrome among the pregnant population of northern shore of Persian Gulf in Iran, were considered.

**Materials and Methods**: Between Jan. 2008 and Dec. 2009, in a nested case control study, pregnant women with preeclampsia (N=198) as cases and healthy (N=201) as controls were enrolled in the study. DNA were extracted from 10 CC peripheral blood and analyzed for presence of factor V Leiden mutation in these subjects. The maternal and neonatal outcomes of pregnancy according to the distribution of factor V Leiden were also compared among cases.

**Results**: In total, 17(8.6%) of cases and 2(1%) of controls showed the factor V Leiden mutation. The incidence of factor V Leiden was typically higher in preeclamptic women than control group (OR: 9.34 %95 CI: 2.12-41.01). There was no difference in incidence rate of preterm delivery< 37 weeks (OR: 1.23 %95 CI: 0.38-4.02), very early preterm delivery<32 weeks (OR: 1.00 %95 CI: 0.12-8.46), intra uterine fetal growth restriction (IUGR) (OR: 1.32 %95 CI: 0.15-11.30 ),and the rate of cesarean section (OR: 0.88 %95 CI: 0.29-2.62 ) among cases based on the prevalence of factor V Leiden mutation.

**Conclusion**: The pregnant women with factor V Leiden mutation are prone for preeclampsia syndrome during pregnancy, but this risk factor was not correlated to pregnancy complications in the studied women.

## Introduction

The preeclampsia syndrome is a precarious condition with a challenge in medical treatment (1, 2). Two different kinds of treatment have suggested facing with this disastrous condition. One of the treatments is considering expectant management and another is placenta delivery. Considering expectant management especially for preeclampsia syndrome with occurrence before 24 weeks of gestation has reported to be associated with high maternal and fetus morbidity and mortality even though in tertiary referral centers. On the other hand, however, placenta delivery is a rescue medical intervention for mother but it threats the neonatal survival despite spending enough medical care in well- developed medical centers due to early preterm delivery and prematurity, consequently (3-6). 

So, it seems to be efficient to find the ways to prevent the occurrence of preeclampsia syndrome during pregnancy especially in early stages. Some new data support from an increased tendency for coagulation in the pathogenesis of this syndrome. The mutation in the amino acid 506 (R506Q) of factor V gene that is known as Leiden is the most common genetic disorder is assigned pregnant women in a prethrombotic state. In this mutation, Adenine in nucleoside 1691 is substituted with guanine, which results to produce an abnormal activated protein C with decreased anticoagulant activity in subjects with this inherited thrombophilic defect (7, 8). 

Recently it was found that women with this variant allele are at increased risk for pregnancy complications such as: preeclampsia, early onset of preeclampsia, very early preterm delivery, intra uterine growth restriction, low birth weight, small for gestational age, early and late recurrent fetal loss, still birth, placental abruption and susceptibility to recurrence of preeclampsia (9-17); however other studies have not support this hypothesis (18-22). 

Therefore, it seems to be important to investigate the role of prethrombotic factors in the etiology of preeclampsia syndrome and also anticoagulant therapy like heparin therapy to prevent the occurrence of this syndrome in vulnerable women. For this reason, we designed this study to find whether the frequency of Leiden mutation is higher in patients with preeclampsia syndrome and this factor is responsible for higher obstetrics complications in these subjects in an Iranian pregnant population.

## Materials and methods

**Subjects**

Between Jan 2008 and Dec 2009, a nested case control study was conducted to assay the correlation between factor V Leiden mutation and preeclampsia syndrome in pregnant women who were attended to Shariati women hospital for the prenatal care or delivery in Bandar Abbas, Iran. In this study, 198 pregnant women with preeclampsia were enrolled as cases and 201 healthy pregnancies were considered as controls that were matched according to the age, ethnicity, and number of gravidity. 

Cases with multiple pregnancy, molar pregnancy, diabetes mellitus, chronic hypertension, renal diseases, and systemic lupus erythematous, past history of hypertension, recurrent abortion or thromboembolic events were excluded from the study. Such exclusion criteria were also set for 198 matched controls.

After completing demographic data sheet as well as exclusion criteria form for each participant, the systolic and diastolic blood pressure were measured as twice at least 6 hour apart by an appropriate-sized cuff. Then, 10 cc blood samples were obtained from all subjects for DNA analysis to assay the presence of factor V Leiden mutation in these two groups.

All women were observed for neonatal and maternal outcomes including gestational age at delivery, preterm delivery <37 weeks, very early preterm delivery <32 weeks, early onset of preeclampsia <30 weeks, intra uterine growth restriction, intra uterine fetal death and also cesarean section or normal vaginal delivery at the end of pregnancy. Finally, the pregnancy outcomes were compared among cases in according to the FV Leiden mutation.

In this study, the criteria from the American college of obstetrics and gynecologists were used to detection preeclampsia syndrome in studied population (23). In brief, systolic blood pressure of 140 mm hg or higher or a diastolic blood pressure of 90 mm hg or higher, association of 300 mg protein in 24-hour urine or ≥+1 on two random urine sample dipsticks at least 6 hour apart and also no more than 1 week apart that typically developed after 20 weeks gestation in women without a past history of chronic hypertension was considered as preeclampsia. 

Preeclampsia associated with heavy proteinuria ≥5gr/24hour or ≥+3 on two dipsticks, oliguria ≤500cc/24hour, platelet count ≤100'000, elevated liver enzymes with persistent epigastric or right upper quadrant pain, pulmonary edema, blood pressure ≥160/110, severe headache or visual disturbance recognized as severe preeclampsia. Eclampsia is defined as occurrence of seizure without any identifiable etiology in preeclamptic women and Hellp syndrome as hemolysis, elevated liver enzymes and low platelet counts in studied patients. 

Pregnant women with Hellp syndrome or eclampsia was classified as sever preeclampsia. In this study, we recorded IUGR as child birth weight <10 the percentile for sex and gestational age based twice sonography 2 weeks apart. The gestational age was measured using last menstrual period (LMP) and if it was unknown, we used ultra sonography instead. The ethics committee of Hormozgan University of Medical Sciences approved the study and signed informed consent was obtained from all subjects to participate at the study.

**Molecular Study**

To determine of FV Leiden mutation**, **Genomic DNA extraction was performed following protocol described by Miller *et al* (24). PCR amplification was carried out in 25 l reaction volume containing 0.5 units Taq polymerase, 200 µM dNTP, 500 µM of each of previously described primers. After initial denaturation, 35 cycles at 95C for 30s, 57C 30s, and 72C for 20s and followed extension by 72 for 10 min were performed. About 10 µl of PCR product digest with MNl I restriction enzymes (25) and result was analyzed by agarose gel electrophoresis containing ethidium bromide to find Wild Genotype: G/G, Mutant Genotype (Heterozygote): G/A and Mutant Genotype (Homozygote): A/A. (G=guanine, A=Adenine)

**Statistical analysis**

The SPSS software (version13, Inc, Chicago, Illinois, USA) was used to analysis the collected data. The chi-squared test was applied to determine the correlation between factor V Leiden mutation and preeclampsia in this study and also we performed t-test to compare quantitative variables between cases and controls. The correlation between Leiden mutation and the incidence rates of IUGR and IUFD were examined by Fisher's Exact Test. A p-value<0.05 was considered significant statistically and odds ratio (OR) with %95 confidence interval was calculated for pregnancy complication variables on Iranian population curve and we observed IUFD as not detectable fetal heart rate in at least 

## Results

Three women who met the exclusion criteria were drawn from the study cases. The age, race, and number of gravidity were not different among cases and controls. Duration of pregnancy was lower in patients than control group that it was expectable. The demographic data and clinical characteristics of study patients are presented in table I. The prevalence of factor V Leiden was higher in cases 17 (8.6%) than control group 2 (1%) that it was statistically significant (OR: 9.34 %95 CI: 2.12-41.01).

Table II shows the frequency of normal genotype and genotypes with mutant allele in cases and controls. Of the 46 (23.2%) studied women as sever preeclampsia, 3 patients had Hellp syndrome and 6 of them were referred to the hospital with eclampsia. Leiden mutation was only found in 3 patients with severe preeclampsia. There wasn’t any association between severity of preeclampsia and factor V Leiden mutation (OR: 0.68 %95 CI: 0.18-2.50). 

The maternal and neonatal outcomes among the carriers of factor V Leiden are summarized in table III. The frequency of preterm delivery<37 weeks (OR: 1.23 %95 CI: 0.38-4.02) and very early preterm delivery<32 weeks (OR: 0.83 %95 CI: 0.10-7.03) were not higher in women with Leiden mutation than other cases without the mutation. Nine cases delivered IUGR that the majority of them 7 (77.8%) had sever preeclampsia. Three cases with wild type factor V were found with IUFD. The incidence rates of IUGR and IUFD among cases were not correlated to the presence of factor V gene mutation (Table III). 

Of the 198 cases studied, 135 (68.2%) had normal vaginal delivery and 63 (31.8%) underwent cesarean section. In patients with cesarean section, 5 (2.5%) had factor V mutation and rest of them were negative for the mutant allele 58 (29.3%). The mode of delivery between cases based on Leiden mutation was not statistically different (Table III). In the present study, the correlation between Leiden mutation and severity of proteinuria as an indicator of the presence of factor V Leiden was examined but it was not significant (Table III).

**Table I:** Demographic data of cases and controls

	**Cases** **n=198**	**Controls** **n=201**	**p-value**
Maternal age (years)	25.98±5.78 (15-40)	25.98±5.54 (14-40)	0.99
Gestational age at delivery (weeks)	36.22±2.70 (22-39)	37.52±0.96 (34-42)	0.00
Gravidity (mean)	2.28±1.74 (1-10)	2.27±1.45 (1-9)	0.95
Systolic blood pressure (mm hg)	147.67±11.02	109.43±10.56	0.00
Diastolic blood pressure (mm hg)	96.87±8.47	72.81±7.44	0.00

**Table II:** Frequency of normal genotype and genotypes with mutant allele in studied patients.

**Studied population genotype**	**G/G (n%)**	**G/A (n%)**	**A/A (n%)**
Preeclampsia women (n=198)	181 (91.4%)	17 (8.6%)	0 (0%)
Controls (n=201)	199 (99%)	2 (1%)	0 (0%)

Wild genotype: G/G

Mutant genotype (Heterozygote): G/A

Mutant genotype (Homozygote): A/A

**Table III:** Maternal and neonatal outcomes according to the presence of FV Leiden mutation among cases

**FVL Mutation**	**Yes (n%)**	**No (n%)**	**p-value**	**Odds ratio (%95 CI)**
Gestational age at delivery (weeks)	36.12±2.69	36.23±2.71	0.87	-
Preterm delivery < 37 weeks	4 (2%)	36 (18.2%)	0.72	1.23 (0.38-4.02)
Very early preterm delivery <32 weeks	1 (0.5%)	9 (4.5%)	0.87	0.83 (0.10-7.03)
IUGR	1 (0.5%)	8 (4%)	0.56	1.32 (0.15-11.30)
IUFD	0 (0%)	3 (1.5%)	0.76	-
Cesarean section	5 (2.5%)	58 (29.3%)	0.82	0.88 (0.29-2.62)
Early onset of preeclampsia < 30 weeks	0 (0%)	9 (4.5%)	0.34	-
Sever preeclampsia	3 (1.5%)	43 (21.7%)	0.56	0.68 (0.18-2.50)
Proteinuria ≥ +3 or more	2 (1%)	19 (9.6%)	0.87	1.13 (0.24-5.35)

## Discussion

The role of genetic factors in the etiology of preeclampsia is not confirmed yet. However in recent decades, several studies have shown that pregnant women who are carriers of factor V Leiden mutation have faced with an increased risk of preeclampsia in their pregnancy but other studies have not support this hypothesis. Some studies reported the prevalence of factor V Leiden mutation varies among a nation to other nation and it's observed the lowest prevalence of the mutation among Asian nations especially in Indonesian and Japanese population (26-29). 

It is notable to mention that there are few studies that have assessed the frequency of Leiden mutation among Iranians. In one study on Kurdish population in western Iran, it was reported the prevalence of 2.97% in 434 healthy subjects and in another study in South of Iran by Karimi *et al* it was obtained 4.1% among 198 volunteers healthy and 6.42% in Kuwaitis of Iranian origin by Dashti *et al* (30-32). 

The present study investigated whether the factor V Leiden mutation is able to predispose pregnant women to preeclampsia and also to alter the pregnancy outcome in these subjects in Iran where is located in the western Mediterranean region and to our knowledge, there are only one study with a very small number of cases (33) that have assessed this correlation in this region of the world.

The overall prevalence of Leiden mutation in preeclamptic women was obtained 8.6%, which was comparable with the reported prevalence of 6% (2/33), 8.9% (4/21) and 10% (4/40) in Davalos, Dashti, Dizon-Townson and Prasmusinto *et al* studies, respectively (34-36). In this study, there was statistically a significant correlation between Leiden mutation and the presence of preeclampsia syndrome in Iranian pregnant women. 

This result is in agreement with findings from the several large meta- analysis studies that have explored to determine any significant association between the factor V Leiden mutation and the presence of preeclampsia during pregnancy. In a meta-analysis consist of cohort studies, Dudding *et al* (37) reported that it seems the maternal factor V Leiden mutation is able to increase the risk of preeclampsia until fifty percent levels (50%) and Kosmas *et al *(38) assessed results from 19 studies that have investigated to find this correlation and identified women with this mutation are at increased risk for preeclampsia 2.5 fold higher than women without the mutation. 

In a similar study by Facchinetti *et al *(11), they genotyped 172 cases with a previous history of preeclampsia for common heritable thrombophilic genes mutations and observed women with a thrombophilic gene mutation are susceptible to achieve preeclampsia during their next pregnancy more than 2.5 times higher than controls. In another study by Dalmaz *et al *(39) in Brazilian population, they considered the correlation between methylenetetrahydrofolate reductase (MTHFR), Prothrombin Mutation (FII), Plasminogen activator inhibitor (PAI-1) and also factor V Leiden mutation and mild/sever preeclampsia in 75 pregnant women. 

They inconcluded from the study the presence of these mutations alone are not able to predispose pregnant women for preeclampsia but a combination of them appears to be correlated with the development of preeclampsia syndrome in pregnancy. 

In contrast to our findings, many studies didn’t find any significant correlation between factor V Leiden mutation and increased rate of preeclampsia among pregnant women with this variant allele. Davalos *et al* (34) reported no significant association between factor V Leiden mutation and preeclampsia in 33 Mexican women that compared to 62 normotensive pregnant women as controls. Salomon *et al* (19) study showed no increased rate of preeclampsia in 191 women with common prothrombotic genes compared to 446 controls (4.3% Vs. 5.25 p=0.59). 

Two recent large nested case-control studies couldn’t reveal such association, too. Hiltunen *et al *(6) studied 100000 pregnant women in Finland and found however the presence of factor V Leiden mutation increased the risk of preeclampsia 1.7 fold higher than controls but this association was not statistically significant. In the Montreal preeclampsia study that included 5332 women by Kahn *et al *(40), it was reported no difference in the prevalence of factor V Leiden mutation among preeclamptic women and controls. 

In another interesting survey, two different ethnical nations (Indonesian and Germany/Croatians women) were assessed to clarify any correlation between factor V Leiden mutation and preeclampsia in a case-control study by Prasmusinto *et al *(36), while this correlation was significant in Germany/Croatians women, they couldn’t find such association among Indonesian women because it was not found any women with factor V Leiden in Indonesian cases and control group. The findings from this study imply that the ethnicity plays an important role in the examination such correlation among different nations.

In the current study, the frequency of preterm delivery, very early preterm delivery, early onset of preeclampsia, IUGR, IUFD and also the mode of delivery were not different among cases in according to the distribution of factor V Leiden mutation. The data obtained from the some studies support this finding. In a survey by zahed *et al* (41), they found the factor V Leiden; prothrombin and methylenetetrahydrofolate reductase mutations are not predictable risk factors of disastrous pregnancy outcomes in Lebanese women. 

Salomon *et al* and Dudding *et al *(15, 37) in their studies couldn’t find evidence regarding the higher incidence of IUGR among women with the factor V Leiden mutation while it's reported the IUGR and IUFD were more prevalent in women with the Leiden mutation by Nurk *et al *(9) and in other similar studies (8, 11). Moreover it was observed the higher prevalence of very early preterm delivery (less than 32 weeks) in these subjects in Facchinetti *et al *(7) study contrasting our study.

We expected to observe the high rate of cesarean section in preeclampsia cases with the mutation based on some previous reports that recorded the higher rate of preeclampsia complications among these patients, but it was not significant when compared to cases without the mutation in our study.

The weakness of the study was the small number of studied patients and it was done on a population of Iranian pregnant women that maybe different from other nations genetically, hence other studies with large sample size are needed to examine such association in another ethnical nations.

In the end, it is important to mention however at the present, the role of heritable thrombophilia in the pathogenesis of preeclampsia is not definitely established but a few studies explored that heparin therapy is effective to reduce the adverse pregnancy outcomes in women carrying the V Leiden mutation (5, 42).

In short, the study showed a significant correlation between the occurrence of preeclampsia and mutation in the factor V Leiden that it's necessary to be confirmed by further studies in other areas especially in Iran. The authors emphasize to this important point that the obtained data may be to be different from other nations due to genetic diversity among different nations. 
